# Molecular taxonomic characterisation of *Ancylostoma* spp. and *Strongyloides* spp. in three protected areas in Ghana

**DOI:** 10.3389/fpara.2026.1798958

**Published:** 2026-04-10

**Authors:** Sandra S. Gyarteng Mensah, John Asiedu Larbi, Adrian Streit

**Affiliations:** 1 Department of Integrative Evolutionary Biology, Max Planck Institute for Biology, Tübingen, Germany; 2 Department for Theoretical and Applied Biology, Kwame Nkrumah University of Science and Technology, Kumasi, Ghana; 3 Department of Pharmaceutical Sciences, Sunyani Technical University, Sunyani, Ghana

**Keywords:** *Ancylostoma* spp., Ghana, molecular taxonomy, one health, zoonosis

## Abstract

**Background:**

Soil-transmitted helminths (STHs) continue to affect millions of people worldwide, but the role of animals in sustaining transmission remains poorly understood. This study examined the diversity and zoonotic potential of hookworms and *Strongyloides* across humans, dogs, and nonhuman primates (NHPs) in three different study areas located in three ecological zones in Ghana, where humans live in close contact with domestic dogs and free-ranging nonhuman primates, creating opportunities for cross-species parasite exchange.

**Methods:**

Stool samples from humans, dogs, and nonhuman primates were analysed using the Baermann technique after faecal culture. Single larvae and young adult worms were genotyped at the nuclear small subunit (*SSU*) Hypervariable Regions (HVR) I and IV, ITS-2 and the mitochondrial *cox*-1 markers. Species identities and genetic relationships were explored using Neighbour-joining and Maximum-likelihood phylogenies in MEGA 12 and a Median-joining haplotype network in POpART.

**Results:**

Two major clusters of hookworms were detected in our study area, both belonging to the genus *Ancylostoma*. One cluster is closely related to a species group containing *A. caninum, A. duodenale, A. ceylanicum*, and *A. tubaeform*e. The other cluster appears to belong to the species *A. braziliense*. Several haplotypes in both clusters were shared between humans and dogs, indicating active zoonotic transmission. For *Strongyloides*, humans carried *S. stercoralis*, monkeys carried *S. fuelleborni*, and in dogs we found a *Strongyloides* that appeared closely related to *S. papillosus*.

**Conclusions:**

Our findings reveal substantial hookworm diversity but no evidence for host specificity, which suggests zoonotic transmission at the human-dog interface. The, based on previous literature, unexpected recovery of *A. braziliense* larvae from human stool suggests that the zoonotic potential of this species may be greater than previously assumed. In contrast to several recent studies in Asia, we found no overlap between *Strongyloides* spp. in humans and animals but the number of *Strongylodies* spp. infected hosts was very small. Our results reinforce the need for including animals that live in close proximity to humans in STH studies and control considerations, following a One Health approach.

## 1 Introduction

Soil-transmitted helminths (STHs) are known to pose a significant threat to global health, particularly in tropical and subtropical regions where poverty, poor sanitation, and proximity between humans and animals are common (Pullan and Brooker, 2012; Ng’etich et al., 2024). These intestinal parasites include hookworms, *Strongyloides* spp., *Ascaris* spp., *Enterobius* spp., and *Trichuris trichiura*. Among these STHs, hookworms and *Strongyloides* spp. have a greater impact on human health and can cross species boundaries, contributing to a zoonotic transmission cycle in addition to human-to-human transmission (Fleitas et al., 2020, Fleitas et al., 2022, Pal et al., 2023).

Globally, hookworm infections affect an estimated 780 million people, while *Strongyloides* spp. is estimated to infect around 600 million individuals (De Silva et al., 2003; Bartsch et al., 2016; Buonfrate et al., 2020). These infections hit hardest among children and impoverished communities, leading to anaemia, stunted growth, impaired cognition and increased vulnerability to other infections (Gyarteng Mensah and Larbi, 2025). In particular, hookworms are a significant cause of iron-deficiency anaemia and reduced cognitive development in children, due to chronic intestinal blood loss and nutritional depletion (Stoltzfus et al., 1997; Ellwanger et al., 2022). While most human hookworm infections have been attributed to *Necator americanus* and *Ancylostoma duodenale*, recent molecular epidemiology has identified *Ancylostoma ceylanicum* as an emerging, important species infecting humans (Ngui et al., 2012; Traub, 2013; Samples and Aloh, 2025). In addition, *Ancylostoma braziliense*, *Ancylostoma caninum* and *Ancylostoma tubaeforme*, traditionally parasites of dogs, cats or wildlife, have been documented to cause larva migrans or transient infections in humans (Bowman et al., 2021; Daba et al., 2021). Examples include a 34-year-old French woman who developed cutaneous larva migrans after lying on a sandy beach and was later confirmed by molecular analysis to be infected with *Ancylostoma braziliense* (Le Joncour et al., 2012). Similarly, Tekely et al. (2013) reported a case of a two year old Polish girl who developed cutaneous larva migrans while on vacation in Brazil. Another notable case involved a four-year-old boy living in a rural community in Nepal where both domestic and stray dogs are common. The child was initially misdiagnosed with fungal skin infection, but the lesion continued to spread despite treatment. Further diagnostic investigations confirmed cutaneous larva migrans (Shrestha et al., 2024). These examples highlight the complex ecology of hookworms, showing clearly that humans, domestic animals (such as dogs and cats) and wildlife (including nonhuman primates) can share overlapping parasite pools, creating opportunities for zoonotic transmission.

Similarly, the genus *Strongyloides* spp. is of particular interest for biological and public health reasons. This is because of its unique life-cycle features, such as autoinfection (which can lead to lifelong infection), a free-living generation in the environment, and potential multi-host capability (Page et al., 2018; Carpio and Meseeha, 2023). Although most authors considered the predominant species of *Strongyloides* in humans and in dogs to belong to the species *S. stercoralis*, since the very early days of *S. stercoralis* research, there is an ongoing discussion if the *S. stercoralis* in the two hosts are really the same or just very similar (Fülleborn, 1914; Brumpt, 1922; reviewed in Bradbury and Streit, 2024). Molecular studies suggested that at least two populations of *S. stercoralis* exist in dogs, only one of which is shared between dogs and humans (Jaleta et al., 2017; Nagayasu et al., 2017; Barratt et al., 2019; Beknazarova et al., 2019). Two recent studies, both conducted in Bangladesh, found a “human and dog” type mitochondrial haplotype introgressed into the “dog” only population (based on the nuclear genome), suggesting at least occasional interbreeding of the two (de Ree et al., 2024; Liu et al., 2025). While it appears likely that in most cases human *S. stercoralis* infections are acquired form other humans (Bradbury and Streit, 2024; Liu et al., 2025), dogs and NHP should be considered as reservoir hosts from which infection can reappear in a community after successful treatment (Bradbury and Streit, 2024). *Strongyloides fuelleborni*, which is the predominant *Strongyloides* in old world NHP, is also known to be able to infect humans (Viney et al., 1991; Zhao et al., 2025). Other than *S. stercoralis*, which has been proposed to have spread over the world fairly recently (Nagayasu et al., 2017), *S. fuelleborni* shows a clear geographic population genetic structure with an African and an Asian clade (Barratt and Sapp, 2020; de Ree et al., 2024; Richins et al., 2025). While in Africa cases of *S. fuelleborni* in humans were regularly reported and there is some evidence for human-to-human transmission, in Asia human *S. fuelleborni* were considered exclusively zoonotic and restricted to people with very close contact with NHPs (Barratt and Sapp, 2020; Zhao et al., 2025). However, recent studies in Asia suggested that human *S. fuelleborni* infections might be more common than previously assumed also on this continent (Thanchomnang et al., 2017; de Ree et al., 2024; Zhao et al., 2025).

A retrospective study conducted by Akenten et al. (2022) revealed that in Ghana, there has been a decline in the prevalence of intestinal parasitic infection over recent decades. Nevertheless, epidemiological studies continue to highlight heavy burdens of soil-transmitted helminths in both rural and urban communities with high infection rates for hookworm (*Necator americanus* and *Ancylostoma* spp.) and *Strongyloides stercoralis* (Akenten et al., 2022; Quarcoo et al., 2025). With respect to the actual numbers of infections, different prevalence studies undertaken in Ghana came to very variable conclusions. For example, a survey in northern Ghana found a hookworm prevalence of 50.6% and a *S. stercoralis* prevalence of 11.6% (Yelifari et al., 2005), while another study in the northern and middle belts found STH prevalence of 19.3%, of which hookworm accounted for 12.1% (Adu-Gyasi et al., 2018). For *Strongyloides stercoralis*, a scoping review reported a wide range of prevalence from 0.1% to 41.1% across Ghana, depending on region, diagnostic method, age group, and setting (Dumevi et al., 2025).

Although it is well documented that the STH represent a significant public health burden, substantial knowledge gaps remain. In particular, little is known in many parts of the world, including Ghana, about the prevalence, geographic distribution, and zoonotic potential of less-studied hookworm species such as *A. ceylanicum, A. caninum*, and *Ancylostoma braziliense*. Many studies still rely solely on microscopy, which is limited in its ability to differentiate morphologically similar eggs. Hookworm eggs from humans, dogs, or nonhuman primates can look nearly identical and are often assumed to belong to *Necator americanus* or *Ancylostoma duodenale*, potentially overlooking zoonotic species (O'Connell and Nutman, 2016). Another gap lies in the lack of simultaneous sampling and molecular characterisation across humans, domestic animals, and wildlife in the same ecological setting. Ecosystems such as forest edges or protected areas where humans, animals, and wildlife co-exist may facilitate cross-species transmission, yet remain understudied, particularly with molecular tools.

Although genomic research on the genera *Ancylostoma*, *Necator* and *Strongyloides* has significantly advanced our knowledge of their taxonomy, genetic diversity, and evolutionary relationships, there remain issues in public databases, such as NCBI, that present considerable issues for *Ancylostoma* sequence data. Misidentified entries, poor annotation, incomplete records, and the lack of linked voucher specimens all reduce data reliability and hinder large-scale comparative work (Traub et al., 2007; Smout et al., 2013). For hookworms, molecular identification typically uses the internal transcribed spacer regions ITS-1 and ITS-2 of the ribosomal RNA locus as nuclear markers and/or mitochondrial genes, especially the cytochrome c oxidase subunit I (*cox-1*) (Mejías-Alpízar et al., 2024). For *Strongyloides* spp. molecular taxonomy, the hypervariable regions (HVR) I and IV of the small subunit (*SSU*) rDNA gene are most commonly used as nuclear markers, alongside the *cox-1* locus as mitochondrial marker (Zhou et al., 2019b).

This study seeks to investigate the species and genotype composition of hookworms and *Strongyloides* spp. circulating among humans, domestic dogs and nonhuman primates in three different study areas located in three ecological zones (forest-savannah transition zone, guinea-savannah wood land, and costal savannah) in Ghana in order to access the zoonotic transmission potential of these parasites.

## 2 Materials and methods

### 2.1 Study sites

A cross-sectional study was conducted to collect stool samples from humans, dogs, and nonhuman primates in Boabeng Fiema Monkey Sanctuary, Mole National Park, and Shai Hills reserve, which are protected areas in Ghana. These study sites were selected for their differences in ecological zones and rich biodiversity and because in these areas, there is close interaction among humans, monkeys, and dogs.

Boabeng Fiema Monkey Sanctuary (https://boabengfms.org) (forest-savannah transition zone), lies within the latitude range 7°40’00’’N to 7°44’10’’N and the longitude range 1°37’45’’W to 1°42’00’’W at about 360 m of elevation in the Nkoranza North District of the Bono East region and is home to two species of nonhuman primates: *Colobus vellerosus* (black-and-white colobus) and *Cercopithecus campbelli lowei* (Mona monkeys) (Allotey and Wiafe, 2015). Boabeng Fiema Monkey Sanctuary is also known for its cultural and spiritual significance, which contributes to wildlife conservation efforts. Three communities close to the monkey sanctuary were selected for the study: Boabeng (7°43’06.5 “N 1°41’36.4 “W), Bonte (7°44’26.6 “N 1°40’07.5 “W), and Bomini (7°43’37.3 “N 1°39’19.1 “W). Although owning dogs is officially not allowed in the communities surrounding the Boabeng Fiema Monkey Sanctuary, some inhabitants find ways to keep them.

Mole National Park (https://molenationalpark.org) (guinea-savannah wood land) is the largest national park in Ghana and has the widest range of wildlife. The Park covers 4,480 km² of land around 9˚7039˚N and 1˚8034˚W at about 50 m elevation. Wildlife commonly spotted in Mole include warthogs, baboons, patas monkeys, elephants, antelopes, and bushbucks. In the park, interactions among humans, baboons, and warthogs are easily observed. Warthogs and baboons easily have access to the staff and people living in the park house. We selected two communities which were close to the park, including Larabanga (9°13’12.6” N 1°51’30.6” W) and Mognori (9°17’23.5 “N 1°46’25.0” W). Although dog ownership and livestock grazing are restricted within the park boundaries, interactions between humans, dogs, and wildlife occur in buffer zones.

Shai Hills reserve (https://ghanawildlife.org/shaihills.html) (coastal savannah) is an area of about 51km², which lies between latitudes 5°50’00” N to 5°56’00” N and longitudes 0°01’00” E to 0°06’00” E in the Greater Accra region. The wildlife in the reserve includes bushbucks, Olive baboons, green monkeys and cobs. Doryumu community (5°53’45.8 “N 0°01’05.9” E) was the nearest community for conducting this research, where it is known that some inhabitants pay homage to their ancestors every year and interact with the baboons in the reserve. Among these communities, the highest level of close interactions is observed in Boabeng Fiema, followed by Mole National Park and Shai Hills Reserve.

### 2.2 Ethical clearance

Ethical clearance for the study was obtained from the Committee for Human Research Publication and Ethics of the School of Medical Sciences, Kwame Nkrumah University of Science and Technology (ref. No: CHRPE/AP/278/22 and CHRPE/AP/678/22). Approval was also given by the Wildlife Division of the Forestry Commission of Ghana (ref. No: WD/A30/VOL.13/10) for the collection of faecal samples from nonhuman primates. Following the guide lines of the Declaration of Helsinki, the putative participants were informed about the study and from those who volunteered to participate, written informed consent was obtained prior to sample collection. Lastly, before the collection of a faecal sample from the dog’s rectum, oral consent was obtained from the dog owner.

### 2.3 Stool sample collection and analysis

Stool containers with unique identification numbers were provided to participants who consented to the study by staff of the local health care providers, who also collected them. The identity of the individual behind a certain number was not disclosed to the authors of this study. Parasite positive numbers were reported to the local health care providers to enable treatment. For domestic dogs, stool was collected from the rectum by a registered veterinary officer using a lubricated gloved finger (with glycerine for comfort). Stool collection from nonhuman primates was based on non-invasive techniques. Identified troops of nonhuman primates were systematically tracked for 3 hours during early morning activity. Only fresh stools from nonhuman primates that were not contaminated with soil were collected into the stool container. The stool samples were divided into two portions. 2 g were analysed with the formalin-ether sedimentation method and used in a general parasitological survey to be described elsewhere. The rest of each sample was analysed using the Baermann after culture technique, which allows the isolation of live hookworm and *Strongyloides* larvae, as described by Zhou et al. (2019a), with modifications due to the local infrastructural circumstances. The stool samples were mixed with an equal amount of activated charcoal to enhance aeration and larval migration. Depending on the consistency of the stool (some stools were watery and did not need water added), water was added to make the sample moist, not wet. A damp towel was used to cover the samples to maintain humidity. The mixture was incubated at ambient field temperature for 24–48 hours, as incubators were unavailable on site. Empty bottles were used as stands for the funnels and a hole was drilled on the side to provide access to the tube attached to the funnel. A clip was attached to the tube to control the flow. The stool-charcoal mixture was placed on a clean tissue and suspended in water in the funnel for 2 hours. During this period, larvae would have migrated to the bottom of the apparatus. After 2 hours, the worms were harvested in a watch glass and washed twice with clean water.

### 2.4 Single worm lysis

Some of the worms in the clean water were individually picked into a 0.2ml PCR tube with 10 µl of nuclease-free water and stored in a refrigerator overnight before they were transported on ice to the Parasitology laboratory at Kwame Nkrumah University of Science and Technology, where they were stored in a -80°C freezer. The remaining worms were batch preserved in 80% Ethanol, as described previously (Zhou et al., 2019a). The preparation of single worm lysates was done as described by Zhou et al. (2019a) except that the three times freezing before the addition of the 2x lysis buffer was done at -80°C for 20 minutes to 30 minutes for lack of liquid nitrogen. Single worm lysates and single worms in 10 µL nuclease-free water were stored at -80 °C until they were transported, together with the ethanol preserved samples, to the Max Planck Institute for Biology, Tübingen, for further analysis. The transport was on ice. The samples arrived within less than a week, and were stored at -80°C. The specimen in water or ethanol were lysed in Tübingen as described (Zhou et al., 2019a). The lysates were used fresh for PCR or stored at -20°C.

### 2.5 PCR amplification and sequencing of the nuclear *SSU* and ITS rDNA and the mitochondrial gene *cox-1* and phylogenetic analysis

PCR amplification was performed in a total volume of 10 µl, consisting of 1µl of worm lysate as template, 4µl of Dream Taq Green PCR Master Mix (2X) (Thermo Scientific), 5 µl of nuclease-free water and 0.2 µl each of both the forward and reverse primers. Amplification of *SSU* HVR-I, *SSU* HVR-IV, ITS, and *cox-1* was done as described by Zhou et al. (2019b) and presented in **Table 1**. Notice that in **Table 1** of Zhou et al. (2019b), the sequences of the hookworm ITS primers are permuted. This is corrected in **Table 1**.

**Table 1:** Primers used for amplification and sequencing.

Gene region	Primer type	Primer names	Sequence	Annealing temp.	Product size
*SSU* HVR-I (SS, SP, SF) and (HW)	Fw	RH5401	AAAGATTAAGCCATGCATG	52°C	862bp (SS) 883bp (HW)
	Rev	RH5402	CATTCTTGGCAAATGCTTTCG		
	Seq	RH5403 (SS and Hw)	AGCTGGAATTACCGCGGCTG		
*SSU* HVR-IV (SS, SP)	Fw	18SP4F	GCGAAAGCATTTGCCAA	57°C	712bp (SS & SP)
	Rev	18SPCR	ACGGCCGGTGTGTAC		
	Seq	ZS6269 (SS)	GTGGTGCATGGCCGTTC		
ITS-1, ITS-2 (HW)	Fw	NC5	GTAGGTGAACCTGCGGAAGGATCATT	50°C	1095bp
	Rev	NC2	TTAGTTTCTTTTCCTCCGCT		
	Seq	ZS6991 (ITS-1)	GCTGCGTTTTTCATCGAT*		
		NC2 (ITS-2)	TTAGTTTCTTTTCCTCCGCT*		
*cox-1* (SS and SP)	Fw	ZS6985	GGTGGTTTTGGTAATTGAATG	47°C	837bp
	Rev	ZS6986	ACCAGTYAAACCACCAATAGTAA		
	Seq	ZS6990	GGTTGATAAACTATAACAGTACC		
*cox-1* (HW)	Fw	ZS6492	AAACATGAAACCGCGGAAAG	47°C	963bp
	Rev	ZS6989	TCACCACAAACTAATACCCGT		
	Seq	ZS6989	TCACCACAAACTAATACCCGT		
*cox-1* (SP)	Fw	7519SG	CTGTATTAACTGCTCATGCA	55°C	744bp
	Rev	7520SG	ACATCTGGATAATCWACATATTTACG		
	Seq	7521SG	CCAAATACTTCCTTCTTACCAGTCA		

Table modified from Zhou et al. (2019b). SS, *S. stecoralis*; SP, *S. papillosus*; SF, *S. fuelleborni*; HW, hookworm; *SSU*, small subunit ribosomal DNA (nuclear); HVR, hyper variable region; ITS, internal transcribed region of the ribosomal DNA (nuclear); *cox-1*, gene encoding Cytochrome c oxidase subunit I (mitochondrial); Fw, forward primer; Rev, reverse primer; Seq, sequencing primer. *Notice that these two sequences are flipped in Zhou et al. (2019b).

One µl of PCR product was mixed with 1 µl of the corresponding (**Table 1**) sequencing primer (10 mM) and 8 µl of nuclease-free water and submitted to Genewiz Leipzig, Germany, for Sanger sequencing. In some instances, sequencing of the ITS-2 failed and the sequencing was repeated with the forward PCR primer.

The quality of the nucleotide sequences and the presence of potential hybrid or ambiguous peaks were manually checked by examining chromatograms using the Snapgene software (Dotmatics; https://www.snapgene.com). Clean, high-quality sequences were then compared with each other and with selected sequences deposited in the National Centre for Biotechnology Information (NCBI) database using the BLASTn search tool (http://blast.ncbi.nlm.nih.gov/Blast.cgi) followed by phylogenetic analysis. All phylogenetic analyses were conducted in MEGA12 Kumar et al., 2024, using up to seven parallel computing threads for the ITS-2 analysis and eight threads for the *cox-1* analysis. The sequences were aligned with Muscle and the aligned datasets were used for phylogenetic analysis, employing the Neighbour-joining and Maximum likelihood methods with default settings. The reliability of the resulting tree topologies was assessed through 2000 bootstrap replications (Felsenstein, 1985). For the generation of the haplotype networks for the ITS-2 and *cox-1* markers, MEGA 12 Kumar et al., 2024 was used for alignment, DnaSP 6 (Rozas et al., 2017) to define haplotypes, and PopART for median-joining network construction (Leigh et al., 2015). All new sequences were deposited in GenBank under the accession numbers PX917430-PX917447 (*Ancylostoma* ITS-2), PX917456-917460 (*Strongyloides* spp. *cox-1*) and PX928219-PX928237 (*Ancylostoma cox-1*).

## 3 Results

A total of 504 stool samples were collected in Boabeng Fiema Monkey Sanctuary, Mole National Park, and Shai Hills reserve; 274 from humans, 87 from dogs, and 143 from NHPs. Out of these samples, 187 from humans, 87 from dogs and 144 from NHPs were cultured. The remaining samples weighed less than 5g and were used only in a general parasitological survey based on the Formol-ethyl ether sedimentation method that will be published elsewhere, but not included in this molecular study. From the cultures, a total of 568 single worms were lysed for PCR analysis.

### 3.1 Nematode species composition based on single worm *SSU* analysis

PCR amplification and sequencing of the *SSU* HVR-1 region were successful for 496 lysates. These comprised 203 isolates from dogs, 192 from nonhuman primates (NHPs), and 101 from humans. Preliminary taxonomic assignment (**Table 2**) was performed by comparing the resulting 431 bp *SSU* HVR-1 sequences against reference sequences in GenBank using BLAST.

**Table 2:** Preliminary taxonomic assignment of parasitic nematode isolates based on *SSU* HVR-1 sequence similarity.

Host	No of worms	No of individual hosts	Best BLAST hit	GenBank accession of best hit	Sequence identity
Dog	51	9	*Ancylostoma caninum*	MH508247.1	99-100%
Dog	83	11	*Ancylostoma* sp.	MZ681936.1	98-100%
Dog	5	1	*Strongyloides papillosus*	EF066361.1	100%
Nonhuman primates	76	13	*Necator americanus/Oesophagostomuntiacum*	AJ920340.1/LC415112.1	100%
Nonhuman primates	9	3	*Ancylostoma* sp.	MZ681936.1	98-100%
Nonhuma primates	7	2	*Strongyloides fuelleborni*	AB677955.1	99%
Nonhuman primates	15	3	*Oesophagostomum aculeatum*	AB677956.1	98%
Humans	16	1	*Strongyloides stercoralis*	LC536690.1	100%
Humans	58	10	*Ancylostoma* sp.	MZ681936.1	98%

Among the dog-derived isolates, 51 worms from nine dogs showed 99-100% sequence identity to MH508247.1 (annotated as *Ancylostoma caninum*), while 83 worms from eleven dogs exhibited 99-100% similarity to MZ681936.1 (annotated as *Ancylostoma* sp.). Five worms from one dog were 100% identitical to EF066361.1 (annotated as *Strongyloides papillosus*).

Sequences obtained from NHPs revealed a diverse assemblage of strongylid nematodes. Seventy-six worms from thirteen NHPs showed 100% identity to AJ920348.1 (annotated as *Necator americanus*) and LC415112.1 (*Oesophagostomum muntiacum*). In addition, nine worms from three NHPs were 99% identical to MZ681936.1 (annotated as *Ancylostoma* sp.). Seven worms from two NHPs were 100% identical to AB677955.1(annotated as *Strongyloides fuelleborni*). Finally, nine worms from three NHPs were 98% identical to AB677956 (annotated as *Oesophagostomum aculeatum* AB677956). All these species are known NHP parasites in Asia and in Africa. For these species, zoonotic cases have been described in various places, among them Ghana (Blotkamp et al., 1993, Yalcindag et al., 2021).

Human-derived sequences included 16 worms from a single individual that were 100% identical to LC536690.1 (annotated as *Strongyloides stercoralis*), while 58 worms from ten individuals were identical with MZ681936.1 (annotated as *Ancylostoma* sp.).

The other worms found in the human and dog cultured samples were non-parasites closely related to EU196004.1 (annotated as *Auanema rhodensis*) and LC639822.1 (annotated as *Tokorhabditis tauri*). They were presumably contaminants introduced from the ground or by insects that colonized the samples, as it had also been observed by de Ree et al. (2024). In the cultures from the NHPs, which were collected from the ground, we found what are likely contaminating worms, with 99% to 100% sequence identity with parasites of sheep (e.g. AJ920350.1 [annotated as *Trichostrongylus colubriformis*], AJ920341.1 [annotated as *Chabertia ovina*], and MT026695.1[annotated as *Haemonchus placei*]) as well as rats (AJ920340.1 [annotated as *Cyclodontostomum purvisi*]), both of which were present at the sampling spots.

### 3.2 Taxonomic analysis of the hookworms found in this study

Since the *SSU* HVR-1 sequence is not a suitable marker for distinguishing different hookworms, the internal transcribed spacers 1 and 2 (ITS-1, ITS-2) of the nuclear ribosomal locus are frequently used for this group of nematodes (Gasser et al., 2008; Hasegawa et al., 2014). From all worms that the *SSU* HVR-1 analysis had tentatively identified as hookworms, we attempted to amplify the ITS-2 region and the mitochondrial *cox-1* marker.

#### 3.2.1 ITS-2- based phylogenetic and haplotype network analyses

**Figure 1:**
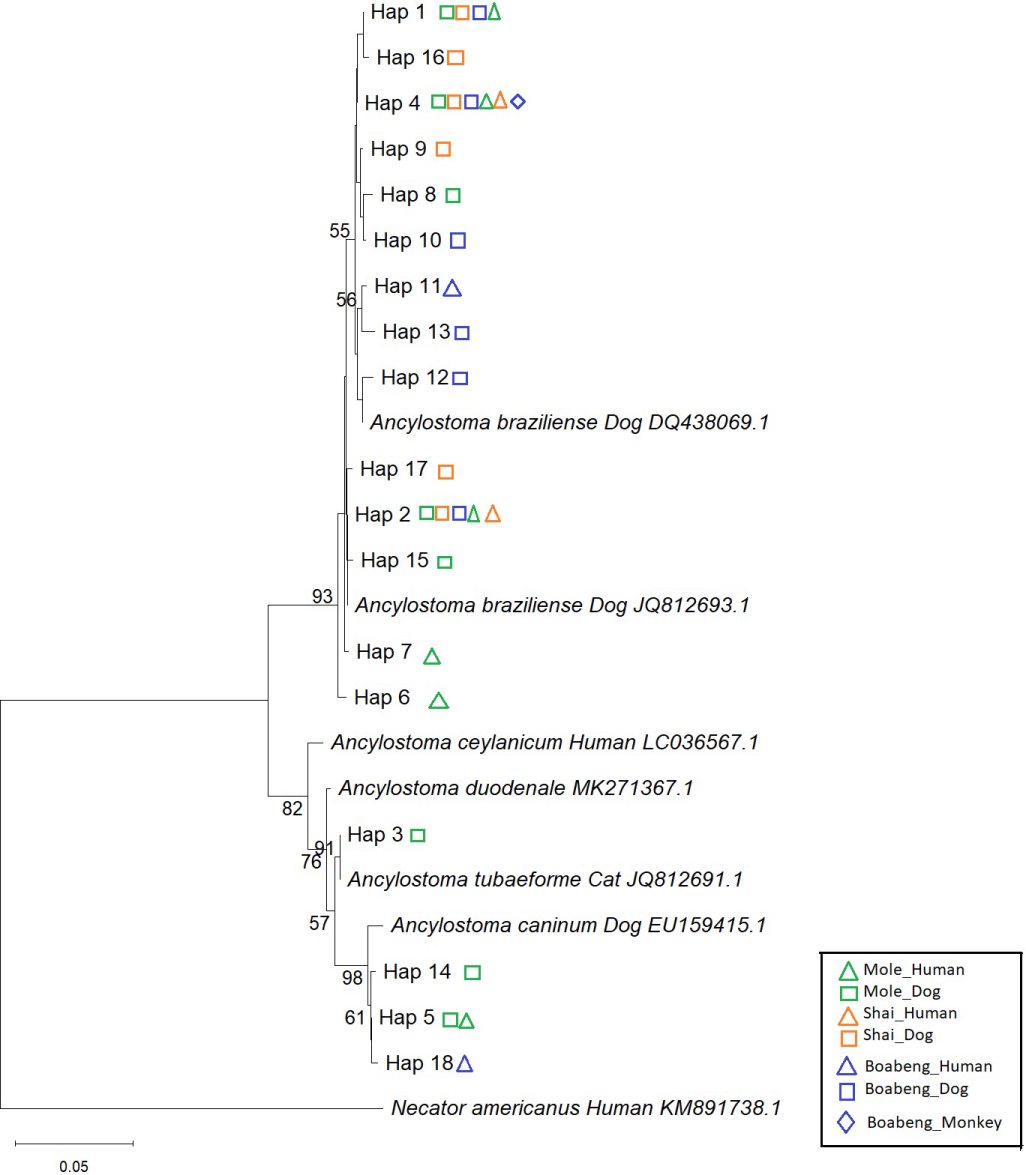
ITS-2 neighbour joining tree of different hookworm isolates. Phylogenetic relationships were inferred using the Neighbor-Joining method (for details see Material and Methods). The analysis included 25 nucleotide sequences corresponding to different haplotypes (Hap). Ambiguous positions were removed using pairwise deletion, resulting in a final alignment of 492 nucleotides. The tree branch lengths are drawn to scale, proportional to the number of base substitutions per site. The scale bar denotes 0.05 base substitutions per site. The branch support values represent bootstrap percentages based on 2,000 replicates and are shown at the corresponding nodes. Only values of 50 and higher are shown. Coloured symbols indicate the place and the host species the particular haplotype was found in. Green: Mole, orange: Shai Hills, blue: Boabeng, triangle: human, square: dog, diamond: monkey.

The ITS-2 sequence was successfully determined from 154 hookworms (44 from humans, 103 from dogs and 7 from NHPs). In total we found 18 different haplotypes (**Supplementary Table 1**). Phylogenetic analysis of the ITS-2 sequences (**Figure 1**) revealed two major clusters, which were both well separated from *Necator americanus*. One *Ancylostoma* spp. cluster, contains worms from all three hosts and all three study areas and also contains the data base entries for *Ancylostoma braziliense*. A smaller number of haplotypes formed the second cluster, together with sequences annotated as *A. duodenale*, *A. ceylanicum, A. caninum* and *A tubaeformae*. Interestingly, within this second cluster, all human derived worms grouped with *A. caninum*, with strong boostrap support.

**Figure 2:**
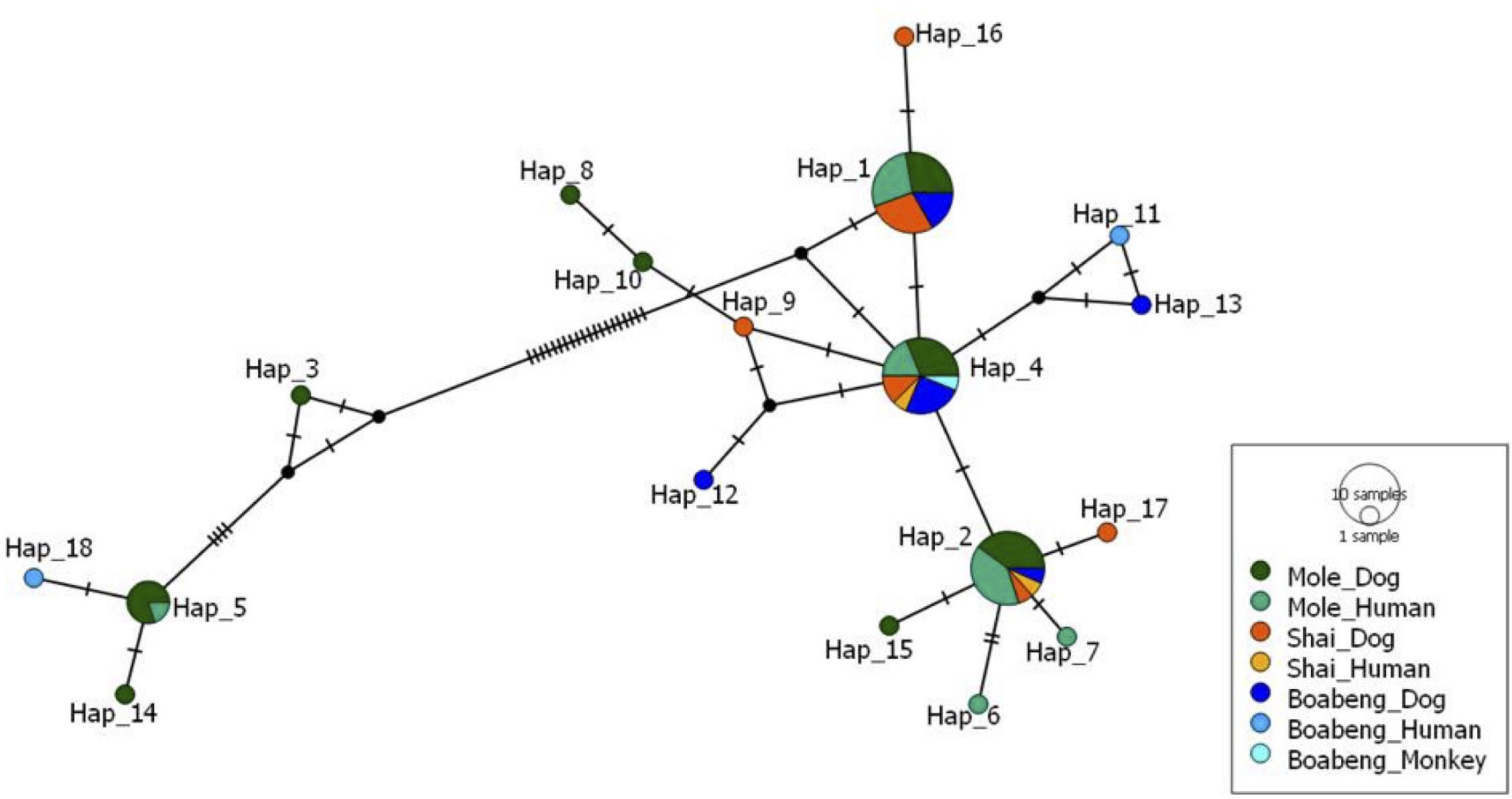
Median-joining haplotype network of *Ancylostoma* isolates based on ITS-2 sequences. For details of the calculation, see Materials and Methods. For the haplotype sequences see **Supplementary Table 1**. The size of the circles indicates how many times the particular haplotype was found. Colours indicate the places and hosts the particular haplotype was found in. Each black bar indicates one mutational change.

The data are also presented in a haplotype network (**Figure 2**). The three most common haplotypes, Hap_1, Hap_2 and Hap_4 were found at all three sampling sites and the four most common haplotypes were found in both humans and dogs.

#### 3.2.2 *cox-1* phylogenetic and haplotype network analyses

**Figure 3:**
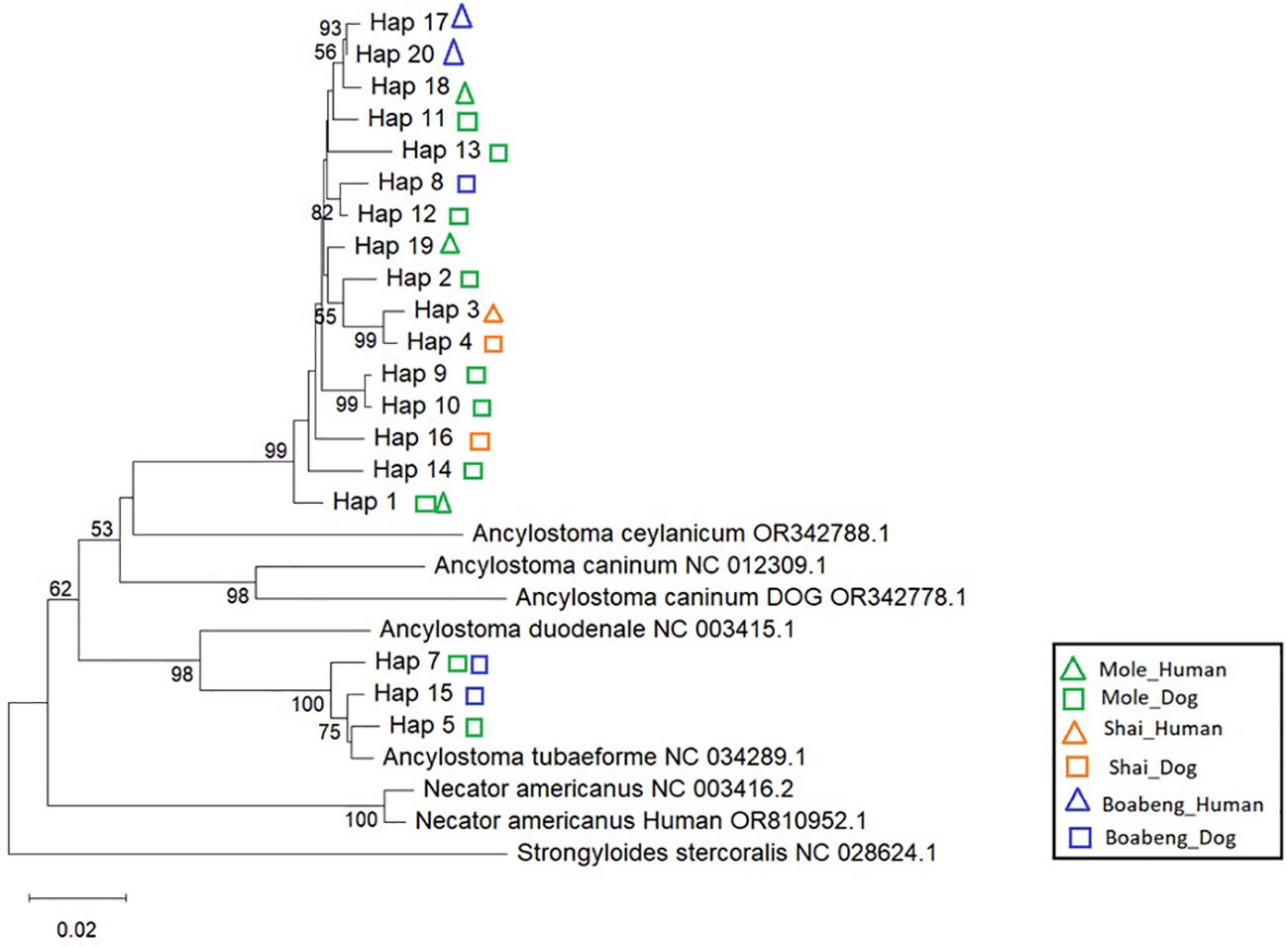
*cox-1* gene tree of different *Ancylostoma* isolates. Phylogenetic relationships were inferred using the Neighbor-Joining method (for details see Material and Methods). The analysis included 27 nucleotide sequences (haplotypes, Hap). Ambiguous positions were removed using pairwise deletion, resulting in a final alignment of 675 nucleotides. The tree branch lengths are drawn to scale, proportional to the number of base substitutions per site. The scale bar denotes 0.02 base substitutions per site. The branch support values represent bootstrap percentages based on 2,000 replicates and are shown at the corresponding nodes. Only values of 50 and higher are shown. Coloured symbols indicate the place and the host species the particular haplotype was found in. Green: Mole, orange: Shai Hills, blue: Boabeng, triangle: human, square: dog.

**Figure 4:**
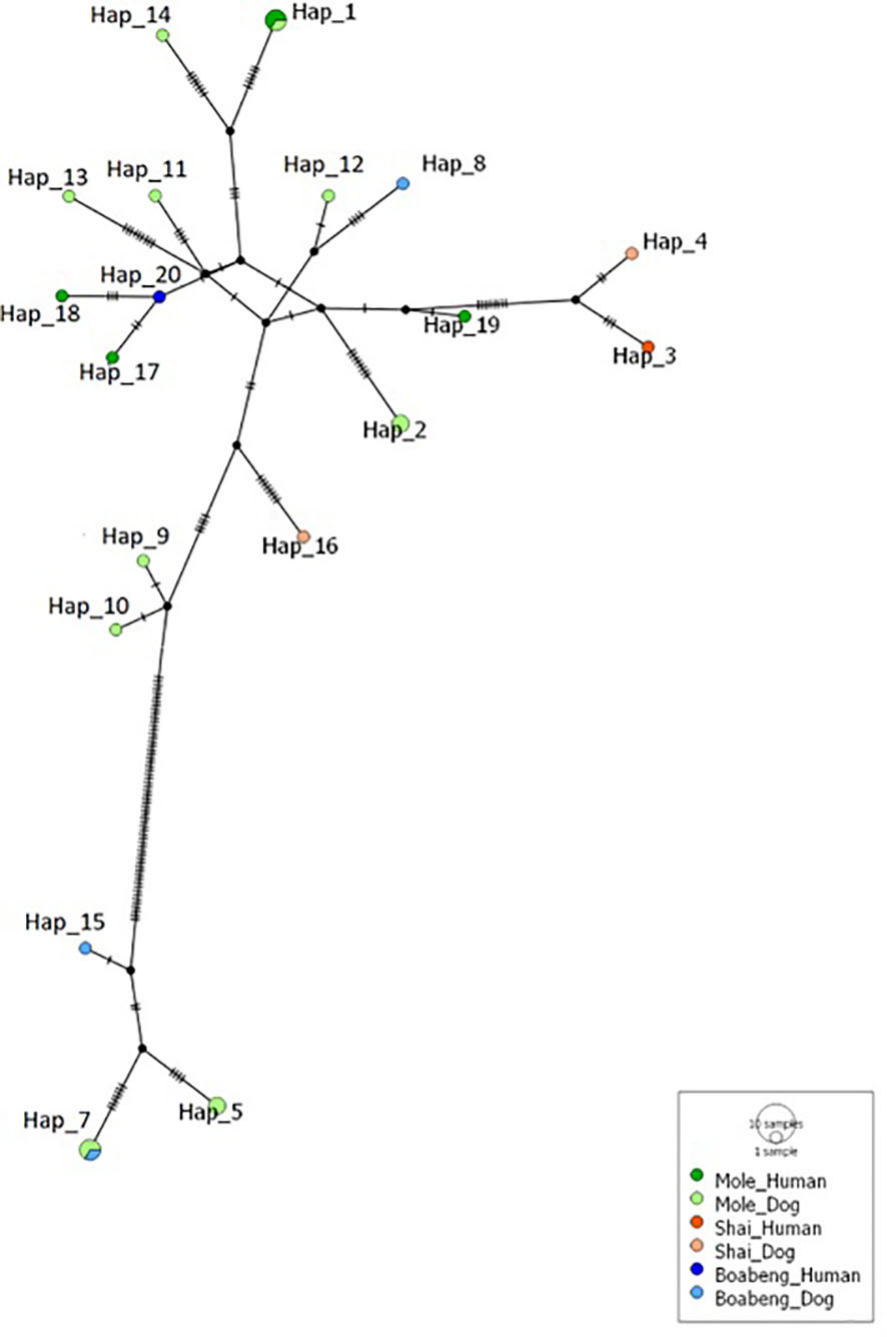
Median-joining haplotype network of *Ancylostoma* isolates based on the *cox-1* gene. For details of the calculation see Materials and Methods. For the haplotype sequences see **Supplementary Table 1**. The size of the circles indicates how many times the particular haplotype was found. Colours indicate the places and hosts the particular haplotype was found in. Each black bar indicates one mutational change.

We found 20 different *cox-1* haplotypes (Hap_1-Hap_20, **Supplementary Table 1**). As with the nuclear ITS-2 sequences, all our hookworm *cox-1* sequences fell into one of two well supported clusters (**Figure 3**). One of them contains worms from all three sites and from humans and dogs. We assume that this cluster represents the species *A. braziliense*, for which, unfortunately, no *cox-1* sequences are in the databases. A smaller number of haplotypes clustered very closely with NC034289.1 (*A. tubaeformae*) and, less closely, with NC003415 (*A. duodenale*). The data are also presented in a haplotype network (**Figure 4**).

### 3.3 *cox-1* phylogenetic tree of *Strongyloides* spp.

**Figure 5:**
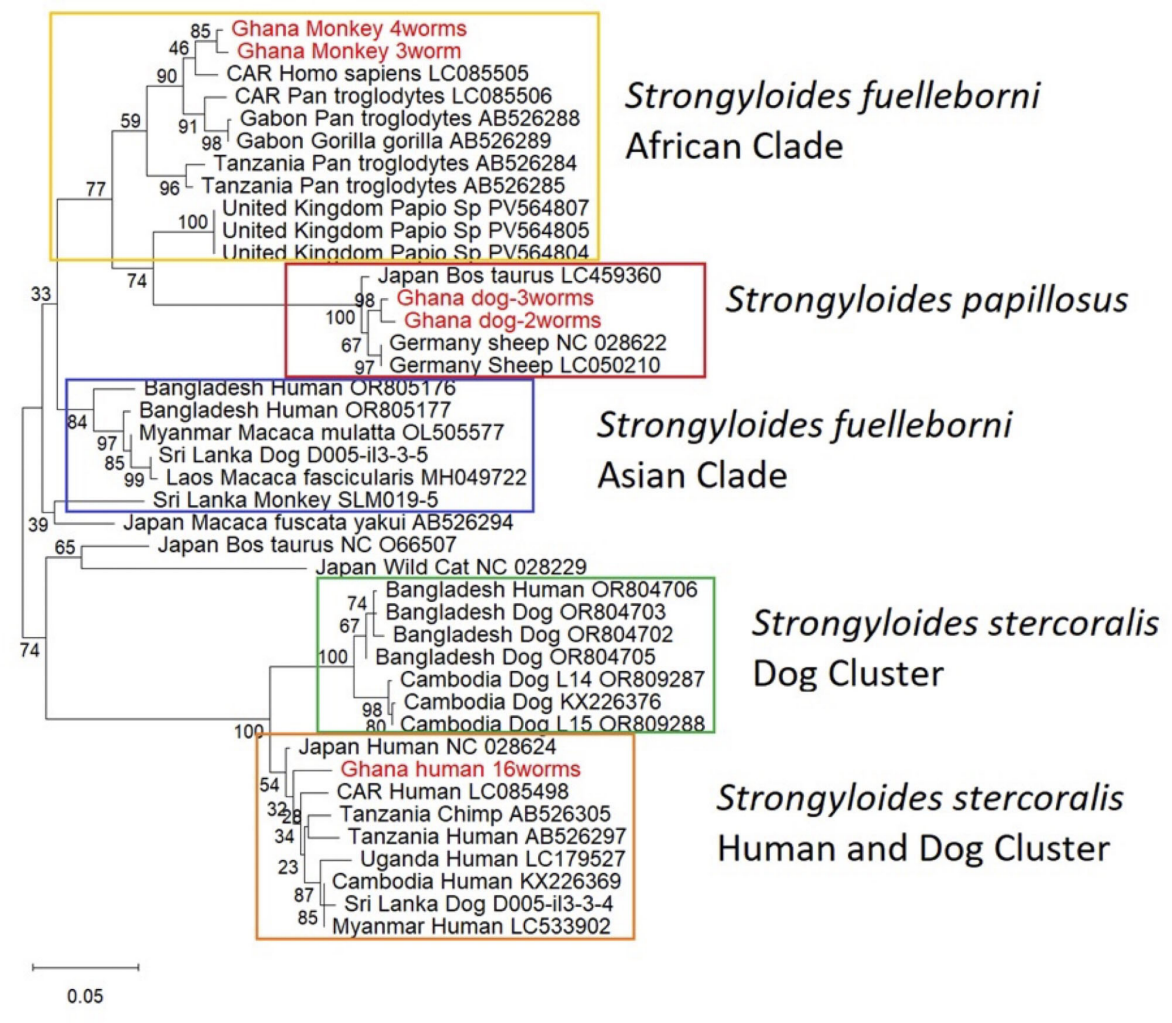
*cox-*1 gene tree of different *Strongyloides* isolates. The phylogeny was inferred using the Maximum Likelihood method with the Tamura and Nei (1993) model of nucleotide substitutions. The tree with the highest log likelihood (-3,425.35) is shown. The analysis included 41 nucleotide sequences. Ambiguous positions were removed using pairwise deletion, resulting in a final alignment of 552 nucleotide sites. The tree branch lengths are drawn to scale, proportional to the number of base substitutions per site. The scale bar denotes 0.05 base substitutions per site. CAR: Central African Republic. Notice that the clustering of *S. papillosus* with the African cluster of *S. fuelleborni* has also been reported by Richins et al. (2025) based on 14 mitochondrial genes.

The number of *Strongyloides* spp. infected hosts we found was very small (**Table 2**). We found two different *cox-1* haplotypes in a total of seven *Strongyloides* spp. from two monkeys, two different haplotypes in a total of five *Strongyloides* spp. from one dog and one haplotype in a total of 16 *Strongyloides* spp. from one human. The *cox*-1 phylogenetic tree (**Figure 5**) revealed three well supported major species-level groupings corresponding to *Strongyloides fuelleborni* (African clade, c.f. Richins et al. [2025], all worms from monkeys), *Strongyloides stercoralis* (all worms from humans) and *Strongyloides papillosus* (all worms from dogs). We cannot know if the dog was indeed infected with *Strongyloides papillosus* or if these worms had just passed through the dog upon eating sheep dung.

## 4 Discussion

To the best of our knowledge, our study is the first study in Ghana that collected samples from humans, dogs and nonhuman primates at the same time and place and it is the first study to provide a molecular taxonomic investigation of hookworms and *Strongyloides* in Ghana.

All hookworms we found and successfully genotyped belonged to the genus *Ancylostoma*. Within this genus, based on the nuclear ITS-2 and the mitochondrial *cox-1* markers they fell into two major groups. We found no evidence for geographic separation or host specificity of the hookworms in our study area, suggesting a largely shared population of hookworms between at least humans and dogs and therefore a large potential for zoonotic hookworm transmission. The number of NHP derived worms was too small to base a solid claim on, but it is noteworthy that all seven NHP derived genotyped *Ancylostoma* sp. showed a haplotype that is one of the most common haplotypes in humans and dogs. We are reluctant to assign species names to the worms we found. Rather little sequence information was determined and the nuclear ITS-2 and in the mitochondrial *cox-1* trees do not agree to which species the smaller cluster is most closely related to. Further, while doing our initial BLAST analysis, we noticed that sometimes identical sequences were annotated as deriving from different species, while different sequences were annotated as representing the same species. Although this is not impossible, we suspect that there are a number of mis-annotations in the databases, as it had been noticed before (e.g. Traub et al., 2007). In spite of these caveats, one of the most notable observations in our analysis is the grouping of the majority of our human and dog isolates and haplotypes (and all monkey derived *Ancylostoma* spp.) within a well-supported ITS-2 cluster that contained only published sequences derived from *A. braziliense*. In the *cox-1* tree the majority of our samples formed a very well supported cluster that did not include any sequences in the databases. There are no *A. braziliense cox-1* sequences in GenBank. By and large, looking at worms for which we obtained the ITS-2 and *cox-1* sequences, this *cox-1* cluster corresponds to the *A. braziliense* ITS-2 cluster but we did find four worms where the nuclear and the mitochondrial markers placed them into different clusters (two in both directions, **Supplementary Table 1**). This may indicate occasional interbreeding between the clusters, but we can also not rule out PCR artifacts, although the negative controls were clean. Our findings suggest an unexpectedly high incidence of *A. braziliense* in our human study population. Previous reports of patent zoonotic hookworm infections in humans primarily involved *A. ceylanicum* and, less frequently, *A. caninum*, both of which are known to have broader host adaptability (Traub et al., 2021; Tenorio et al., 2024). *A. braziliense* is regarded as a hookworm of dogs and cats, with humans serving only as incidental hosts, typically developing cutaneous larva migrans rather than patent intestinal infection (Le Joncour et al., 2012; Daba et al., 2021). *A. braziliense* has even been proposed to be unable to mature within the human gastrointestinal tract and larval or egg shedding in stool has been considered biologically unlikely (Kelsey, 1996; Centres for Disease Control and Prevention, 2019). The fact that we recovered these worms from cultured human stool suggests that they were the products of true infections. It remains to be seen if human infections with *A. braziliense* are a more widespread, although currently under appreciated, phenomenon or if our sampling areas are special cases. In settings like the surrounding communities in Mole and Boabeng-Fiema, characterised by large free-roaming dog populations and heavy environmental contamination, repeated exposure to infective larvae could increase the likelihood of rare intestinal establishment. We cannot completely rule out environmental contamination or the possibility that the larvae were ingested and passed through the gastrointestinal tract without establishing an infection. We consider these possibilities highly unlikely, because fresh faecal samples were collected and ingestion of food contaminated with dog faecal material appears rather unlikely to explain the fairly numerous cases we found.

An important limitation of our study is the rather high number of worms where *cox-1*, and to a lesser extent ITS-2, amplification failed although other markers (*SSU*) worked. We can therefore not exclude that we missed a group of genotypes for which the primers used are not suitable.

The number of *Strongyloides* spp. worms and infected host individuals were small, such that the information that can be gained from them is rather limited. Based on the *SSU* HVR-1 and the *cox*-1 (**Figure 5**) sequences, we identified three *Strongyloides* species circulating in the study areas: *S. fuelleborni* (in a monkeys), *S. stercoralis* (in humans), and *S. papillosus* (in dogs). The first two were expected. The close clustering of the Ghana monkey *S. fuelleborni* isolates with other African *S. fuelleborni* sequences (**Figure 5**) is in agreement with the notion that *S. fuelleborni* is geographically structured with African isolates being genetically distinct from those from Asia (Richins et al., 2025). Our human Ghana isolate of *Strongyloides stercoralis* fell into the human and dog shared cluster (Jaleta et al., 2017; Nagayasu et al., 2017) that contains sequences from Asia, East Africa and Latin America. Compared with *S. fuelleborni*, this reflects the low geographic structuring of human infective *S. stercoralis* proposed by several studies (Barratt et al., 2019;, Aupalee et al., 2020; Barratt and Sapp, 2020; Beiromvand et al., 2024) and supports the notion of Nagayasu et al. (2017) that *S. stercoralis* has spread over the world only rather recently. Finding the sheep parasite *S. papillosus* in dogs was at first surprising. This finding may be related to the fact that dogs and livestock share space in many rural Ghanaian communities such that there may be opportunistic *S. papillosus* infections in dogs or the *S. papillosus* may have passed through the dog’s intestine after eating ruminant dung. These results are consistent with paleoparasitology research from Siberia, where *S. papillosus* eggs and larvae have been found in archaeological dog faeces. In that study, researchers suggested that the dogs were exposed through eating infected livestock offal and animal remains, rather than through actual infection (Losey et al., 2022).

## 5 Conclusion

The main finding of this study is that in three protected areas in Ghana humans and dogs and, at least in the Boabeng Fiema Monkey Sanctuary, nonhuman primates, share hookworm genotypes. Although further studies are needed to elucidate the transmission dynamics, our study strongly suggests that animals form part of the parasite’s reservoir. Consequentially, as it had been proposed earlier (Tenorio et al., 2024; Winslow et al., 2024), human-only deworming strategies, might not interrupt transmission, because the environments remain contaminated. The fact that the few *Strongyloides* spp. worms we found clustered together according to host species should not be taken as evidence for the absence of zoonotic transmission, because they were derived from only very few host individuals. However, it may support the notion that strongyloidiasis is not normally zoonotic (Bradbury and Streit, 2024; Liu et al., 2025).

## Data Availability

The datasets presented in this study can be found in online repositories. The names of the repository/repositories and accession number(s) can be found in the article/**Supplementary Material**.
